# Supplementary figures and images for: Meioc-Piwil1 complexes regulate rRNA transcription for differentiation of spermatogonial stem cells
Source: eLife. 2025 Jul 24;14:RP104295. doi: 10.7554/eLife.104295 (PMC12289311; doi:10.7554/eLife.104295)

5'ETS

ITS1

7SL

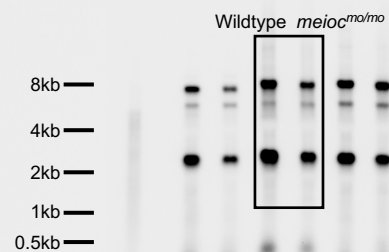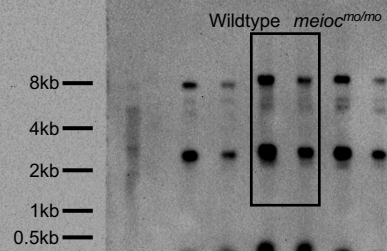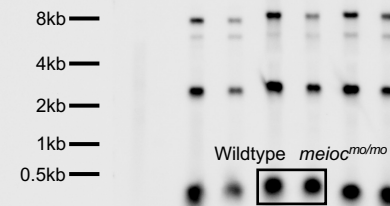

**Figure 2-source data 1.** Original membranes corresponding to Figure 2, panel F.

Supplement: Figure 2—source data 1. [file elife-104295-fig2-data1.zip › Figure 2-source data 1. PDF file containing original northern blots for Figure 2F, indicating the relevant bands and treatments/Figure 2-source data 1 with the relevant bands labelled.pdf]

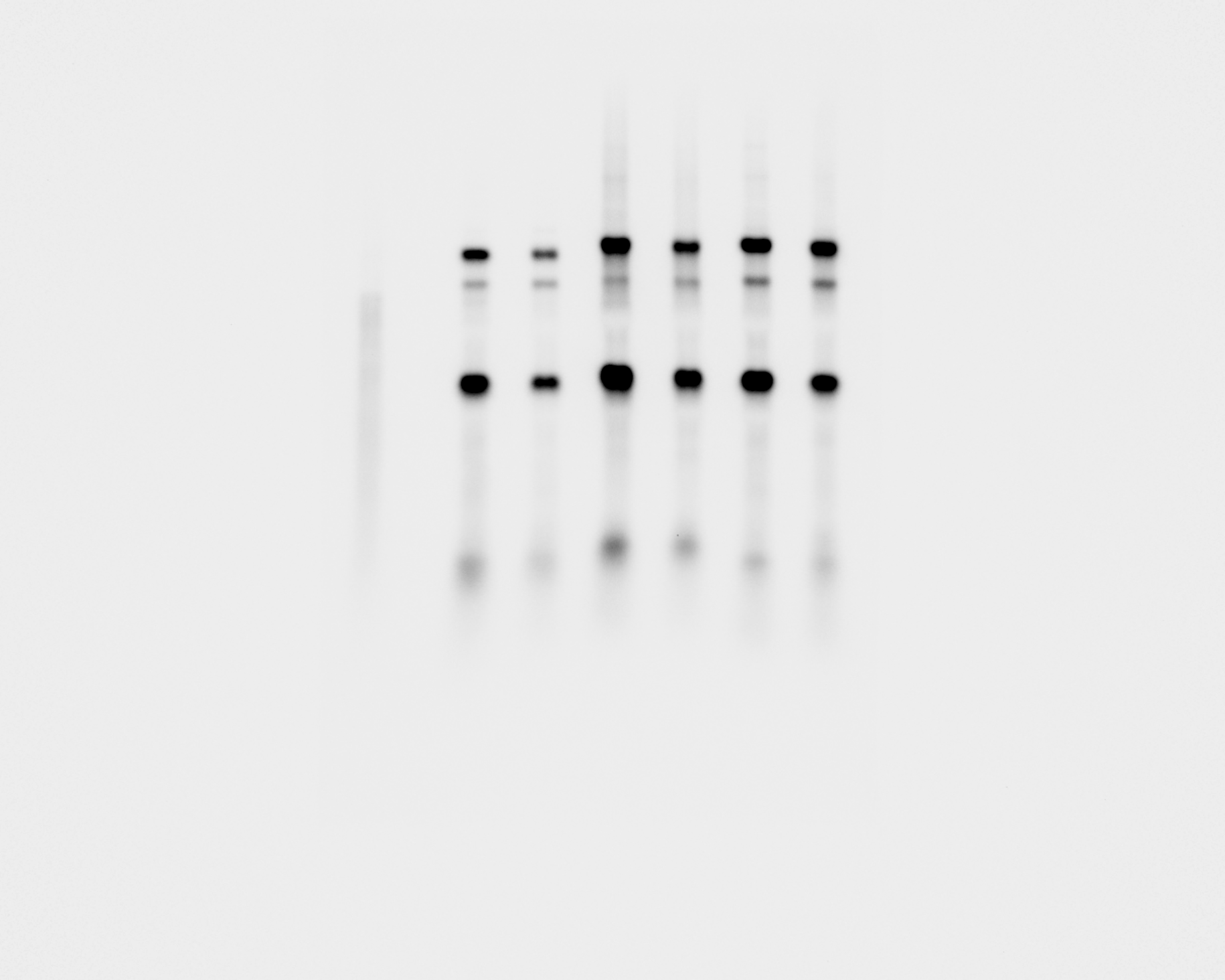

Supplement: Figure 2—source data 1. [file elife-104295-fig2-data1.zip › Figure 2-source data 1. PDF file containing original northern blots for Figure 2F, indicating the relevant bands and treatments/The original file of 5'ETS.tif]

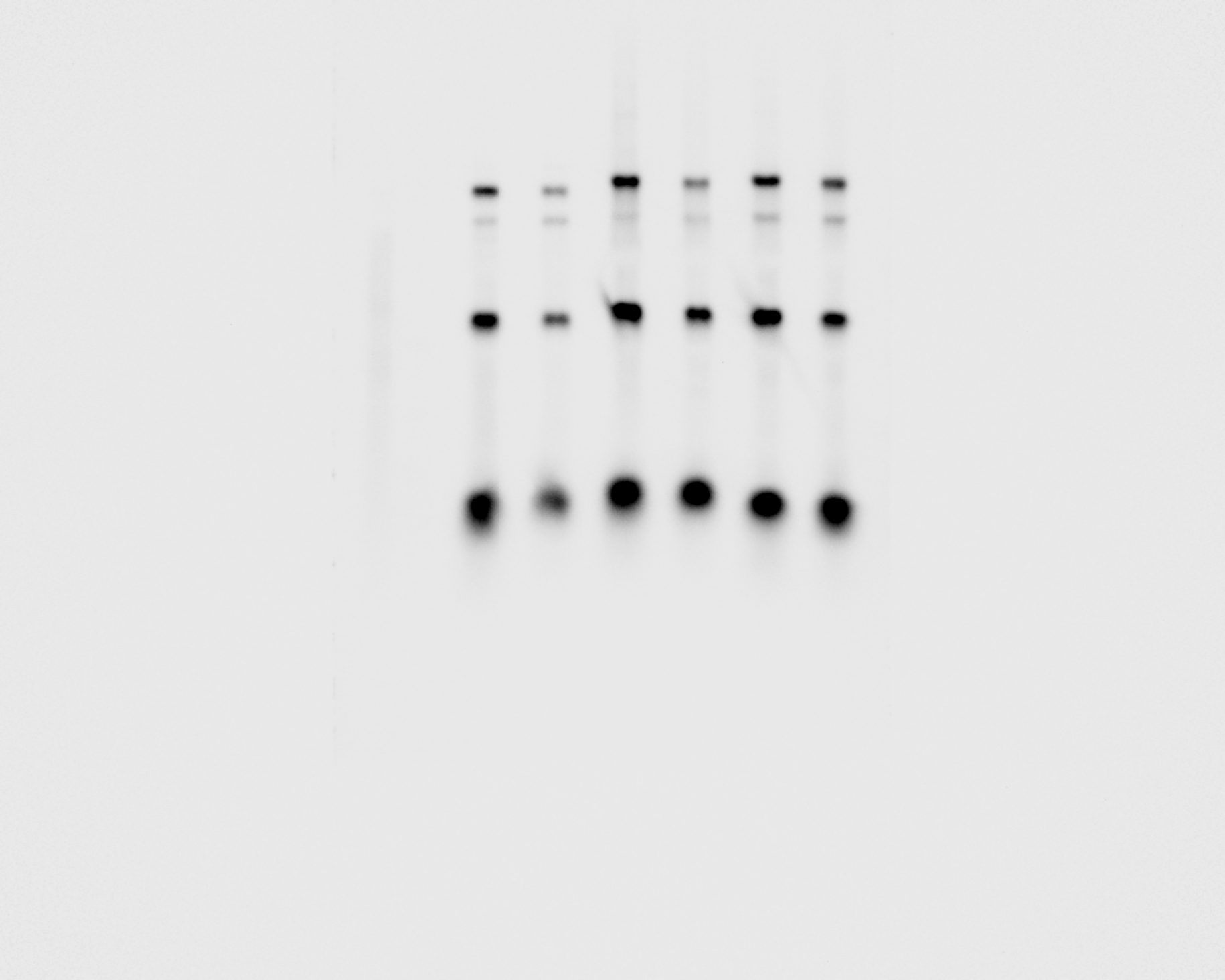

Supplement: Figure 2—source data 1. [file elife-104295-fig2-data1.zip › Figure 2-source data 1. PDF file containing original northern blots for Figure 2F, indicating the relevant bands and treatments/The original file of 7SL.tif]

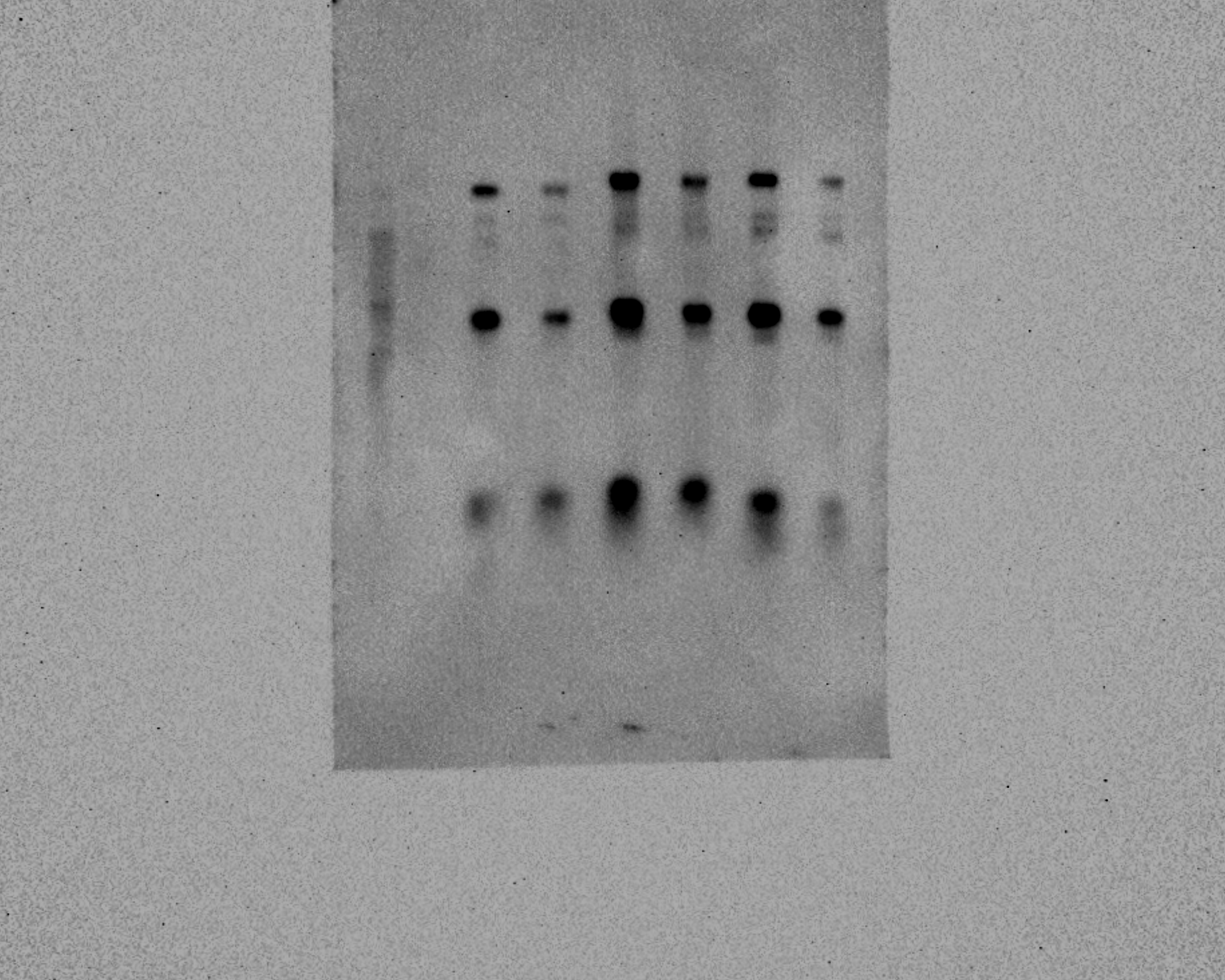

Supplement: Figure 2—source data 1. [file elife-104295-fig2-data1.zip › Figure 2-source data 1. PDF file containing original northern blots for Figure 2F, indicating the relevant bands and treatments/The original file of ITS.tif]

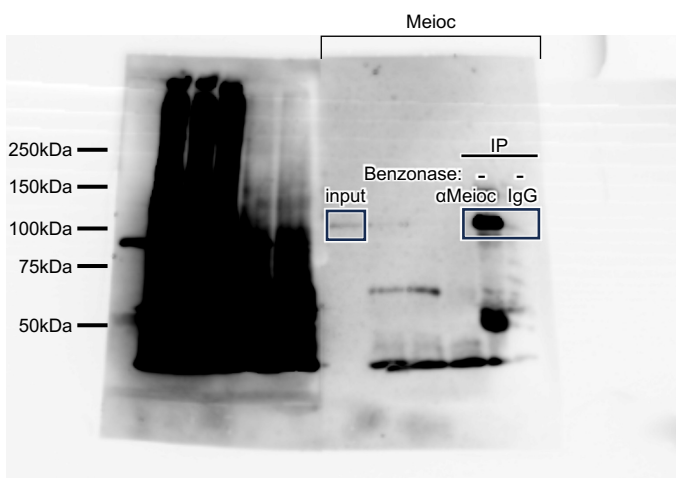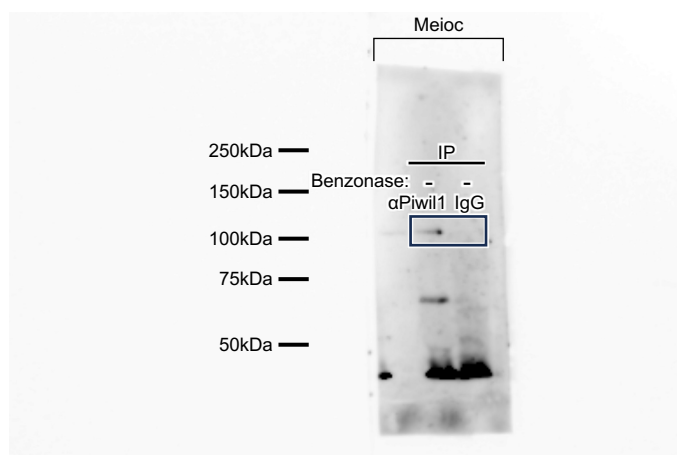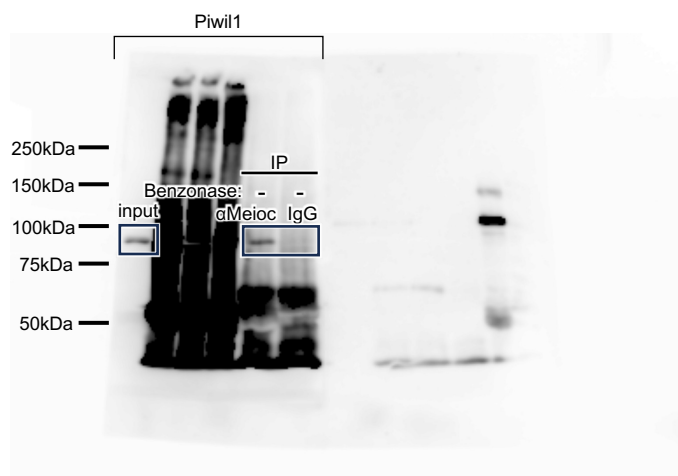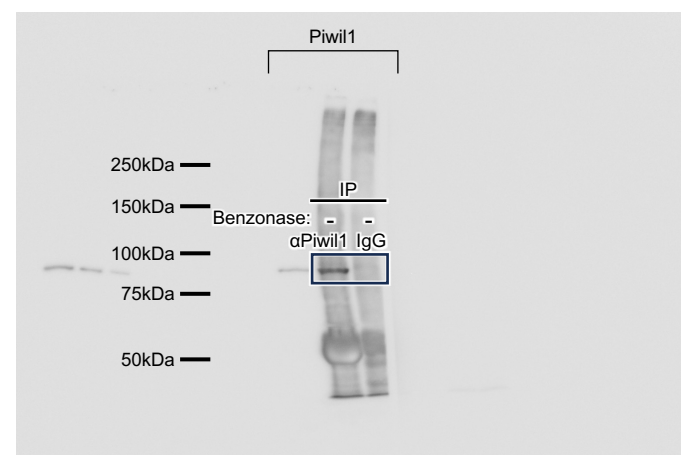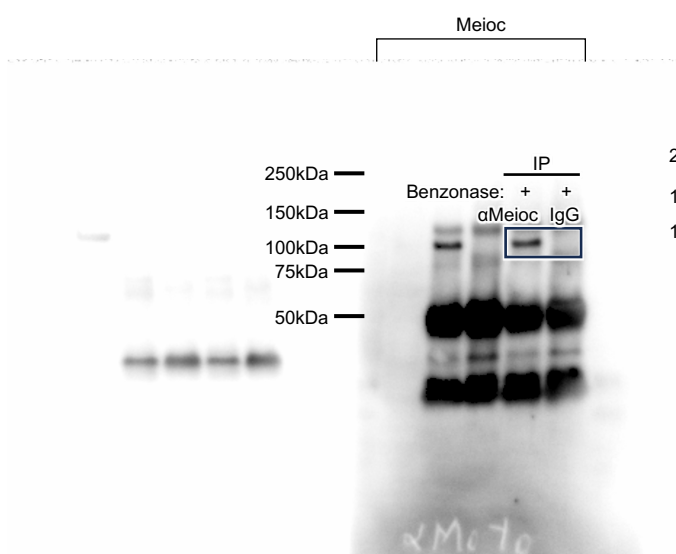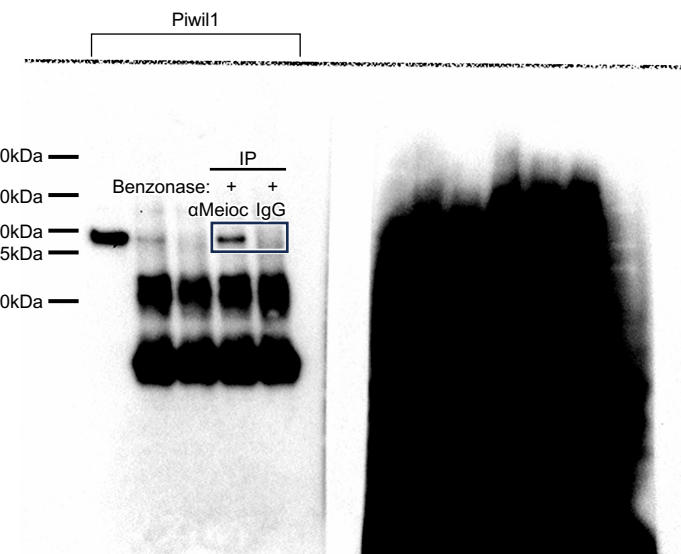

**Figure 4-source data 1.** Original membranes corresponding to Figure 4, panel D.

Supplement: Figure 4—source data 1. [file elife-104295-fig4-data1.zip › Figure 4-source data 1. PDF file containing original western blots for Figure 4D, indicating the relevant bands and treatments/Figure 4-source data 1 with the relevant bands labelled.pdf]

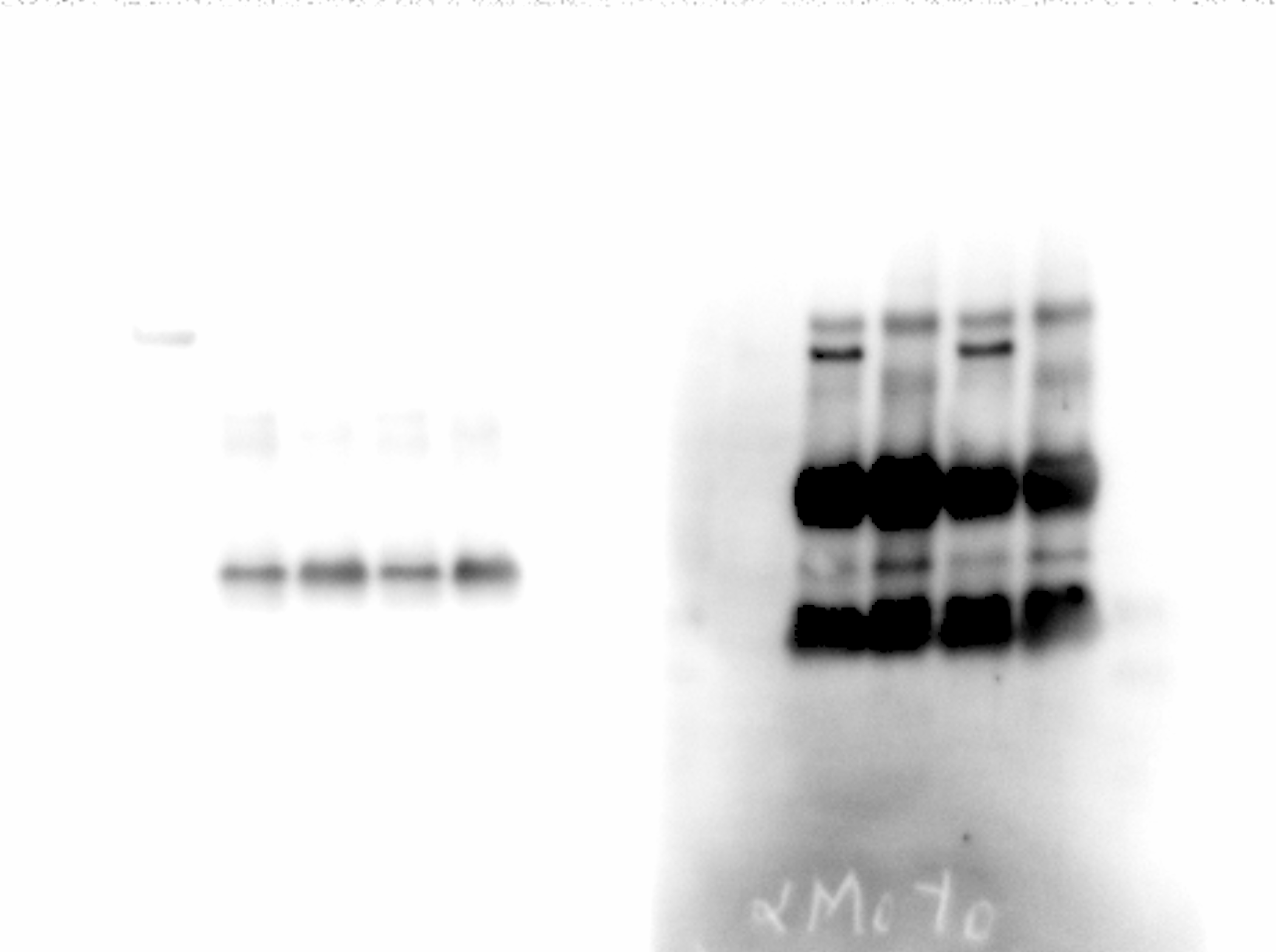

Supplement: Figure 4—source data 1. [file elife-104295-fig4-data1.zip › Figure 4-source data 1. PDF file containing original western blots for Figure 4D, indicating the relevant bands and treatments/The original file of Meioc-WB_Meioc-IP_Bbenzonase.tif]

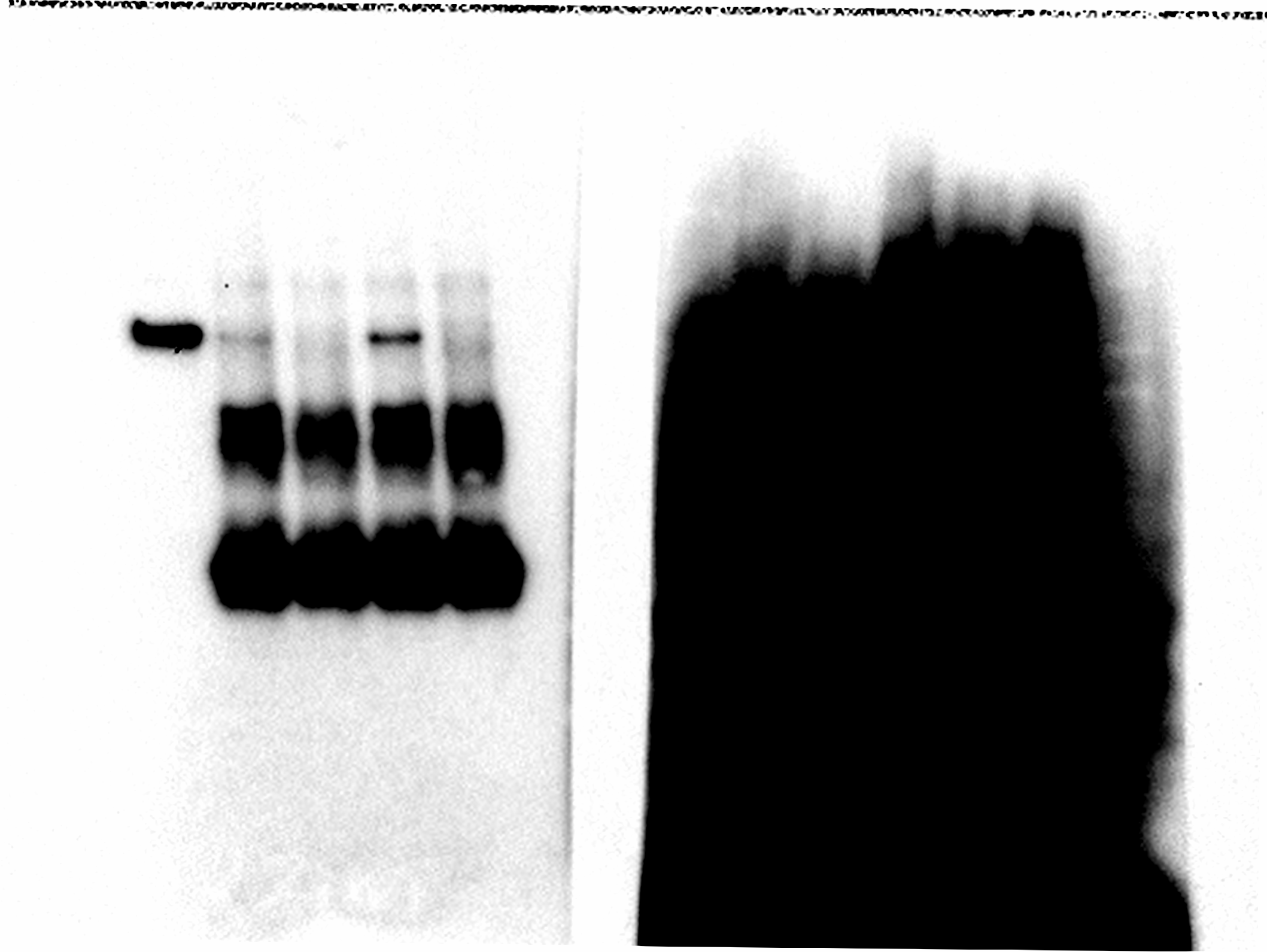

Supplement: Figure 4—source data 1. [file elife-104295-fig4-data1.zip › Figure 4-source data 1. PDF file containing original western blots for Figure 4D, indicating the relevant bands and treatments/The original file of Piwil1-WB_Meioc-IP_Bbenzonase.tif]

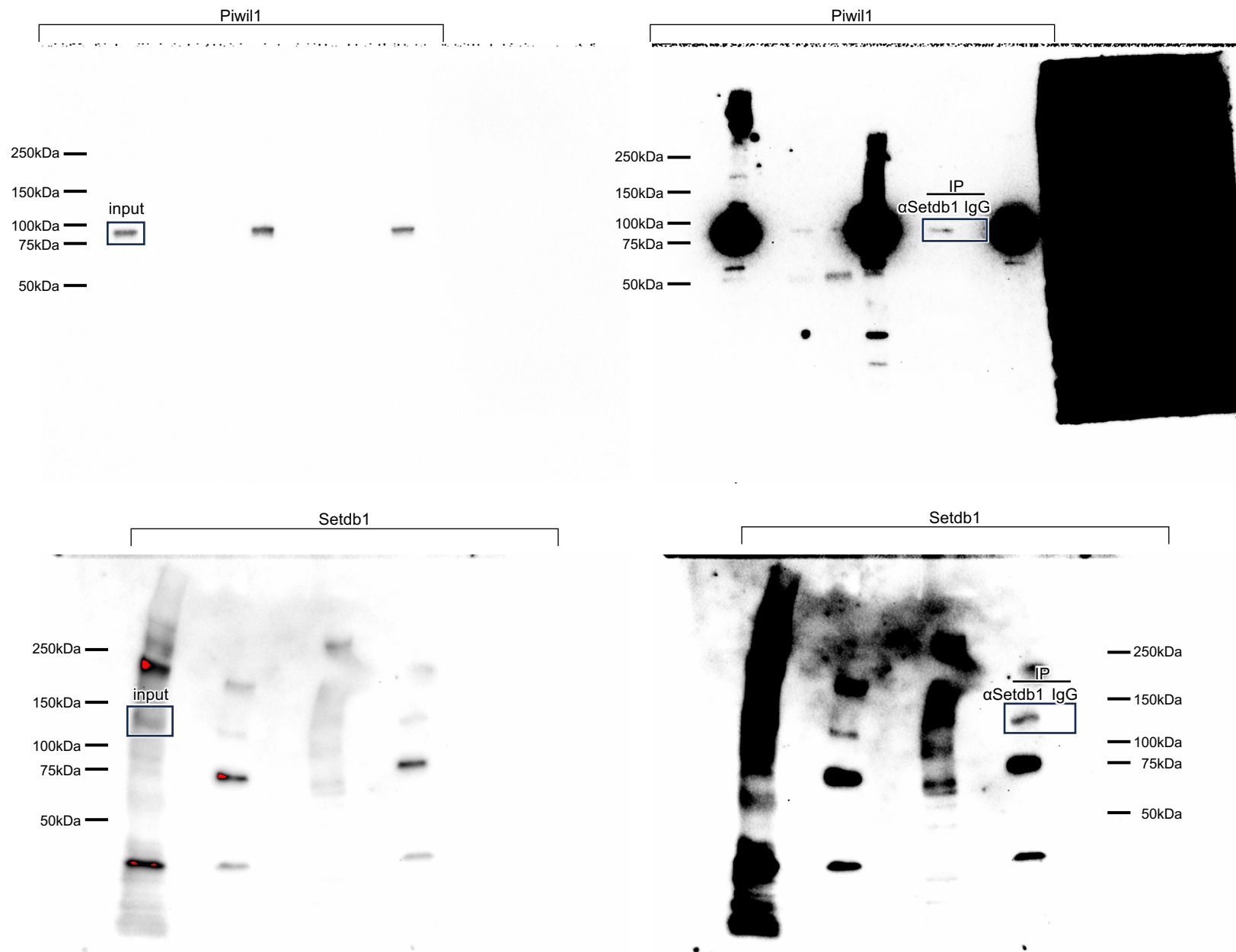

**Figure 6-source data 1.** Original membranes corresponding to Figure 6, panel D.

Supplement: Figure 6—source data 1. [file elife-104295-fig6-data1.zip › Figure 6-source data 1. PDF file containing original western blots for Figure 6D, indicating the relevant bands and treatments/Figure 6-source data 1 with the relevant bands labelled.pdf]

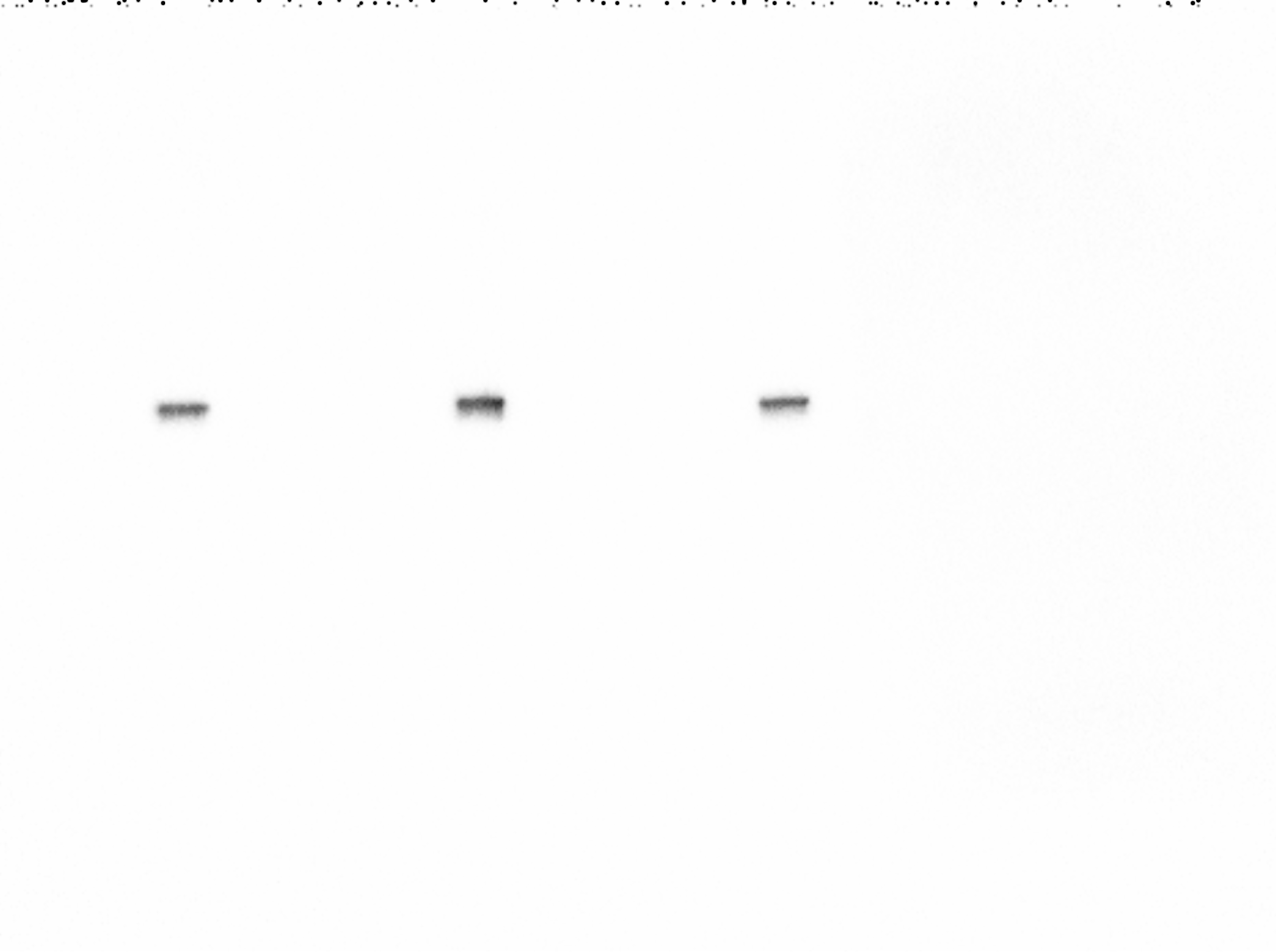

Supplement: Figure 6—source data 1. [file elife-104295-fig6-data1.zip › Figure 6-source data 1. PDF file containing original western blots for Figure 6D, indicating the relevant bands and treatments/The original file of Piwil1-WB_input.tif]

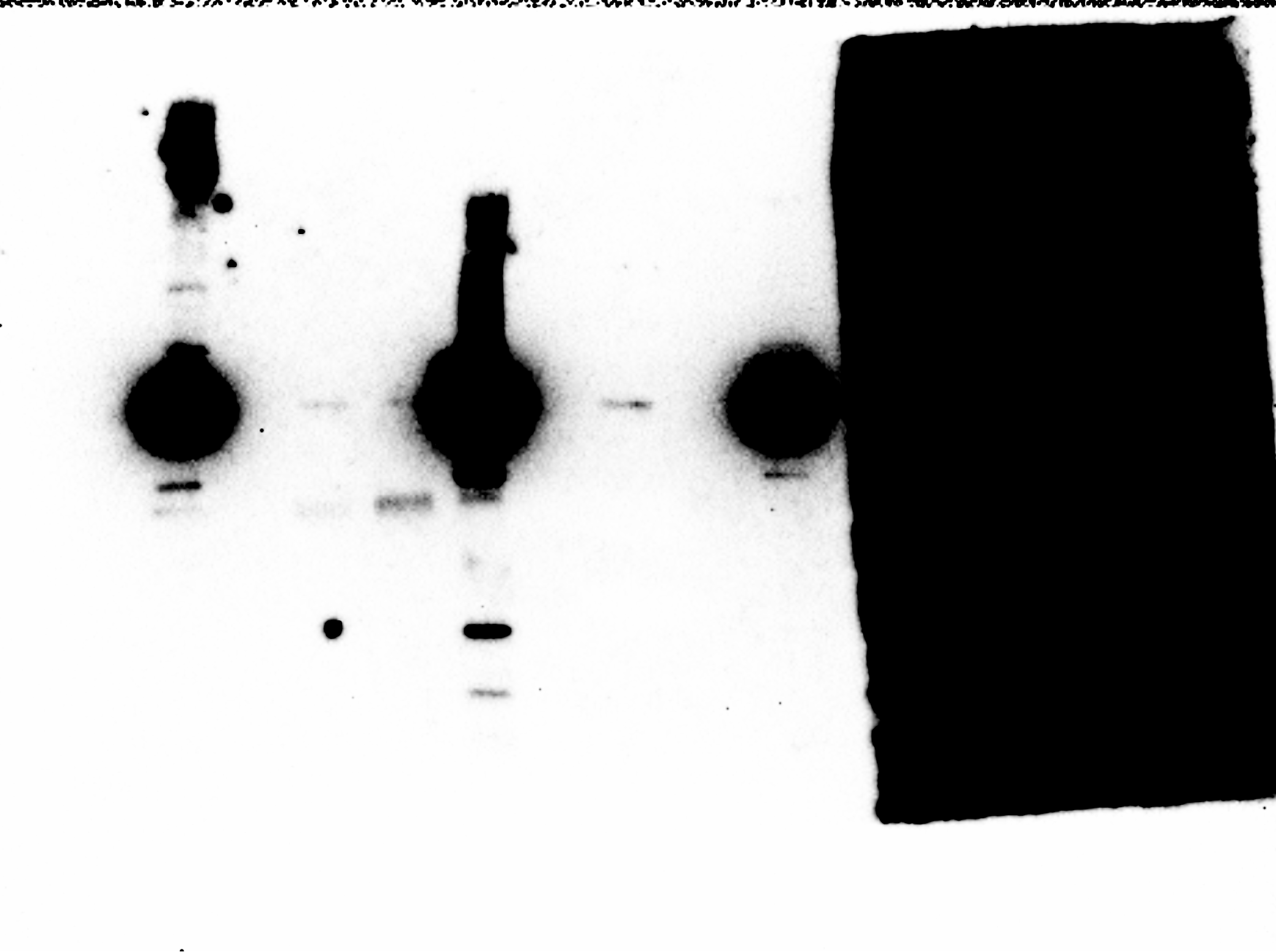

Supplement: Figure 6—source data 1. [file elife-104295-fig6-data1.zip › Figure 6-source data 1. PDF file containing original western blots for Figure 6D, indicating the relevant bands and treatments/The original file of Piwil1-WB_Setdb1-IP.tif]

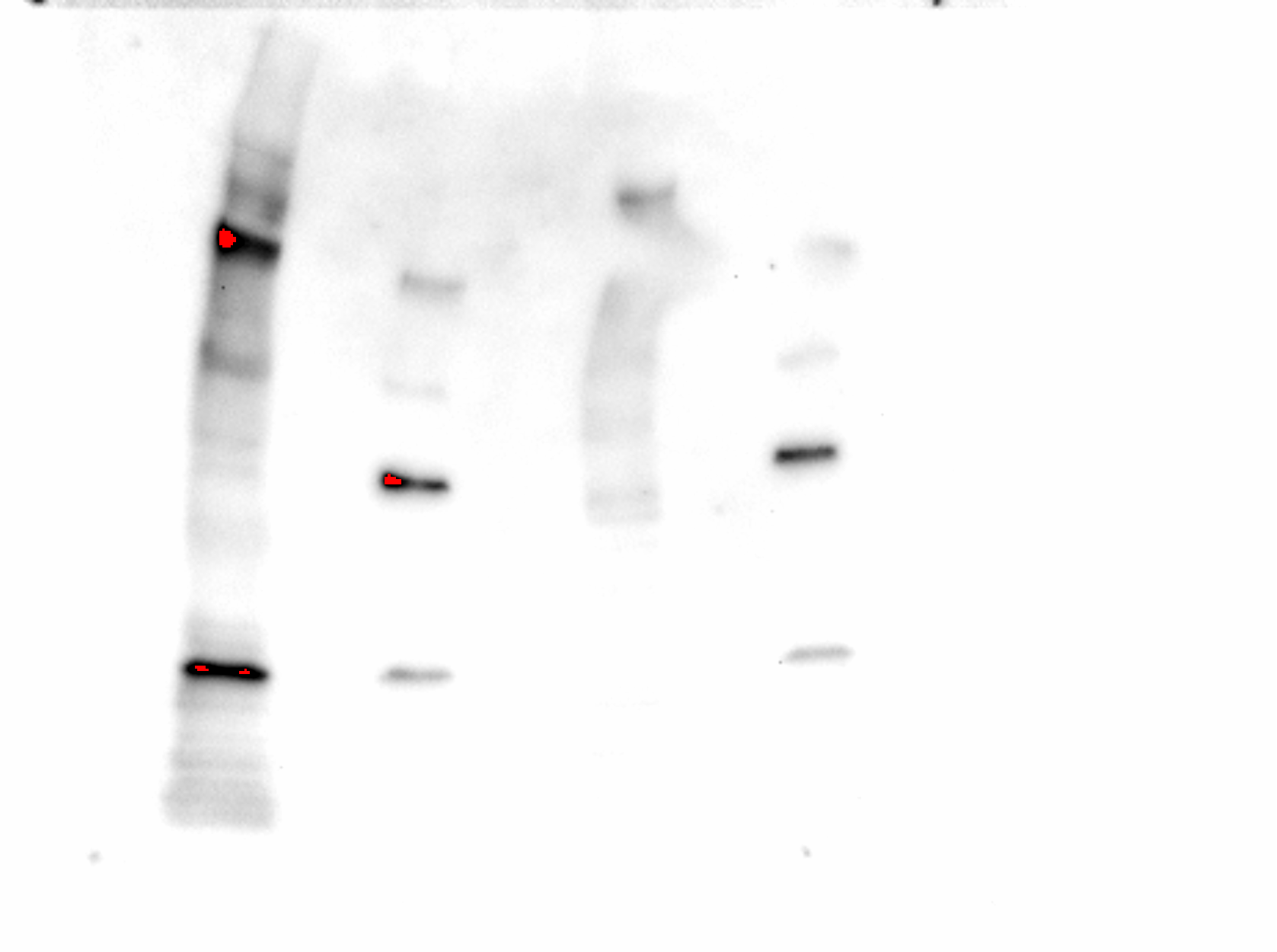

Supplement: Figure 6—source data 1. [file elife-104295-fig6-data1.zip › Figure 6-source data 1. PDF file containing original western blots for Figure 6D, indicating the relevant bands and treatments/The original file of Setdb1-WB_input.tif]

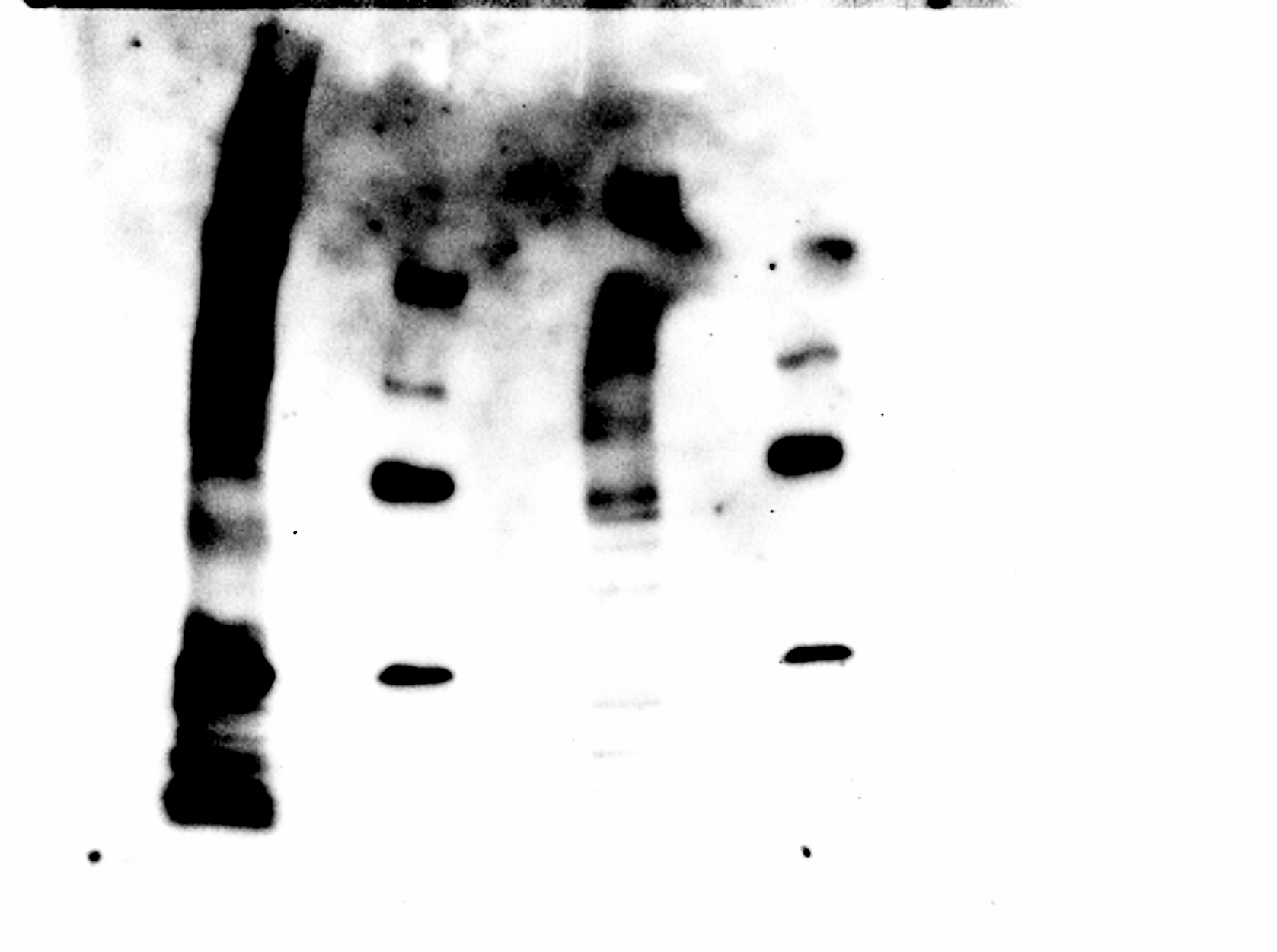

Supplement: Figure 6—source data 1. [file elife-104295-fig6-data1.zip › Figure 6-source data 1. PDF file containing original western blots for Figure 6D, indicating the relevant bands and treatments/The original file of Setdb1-WB_Setdb1-IP.tif]

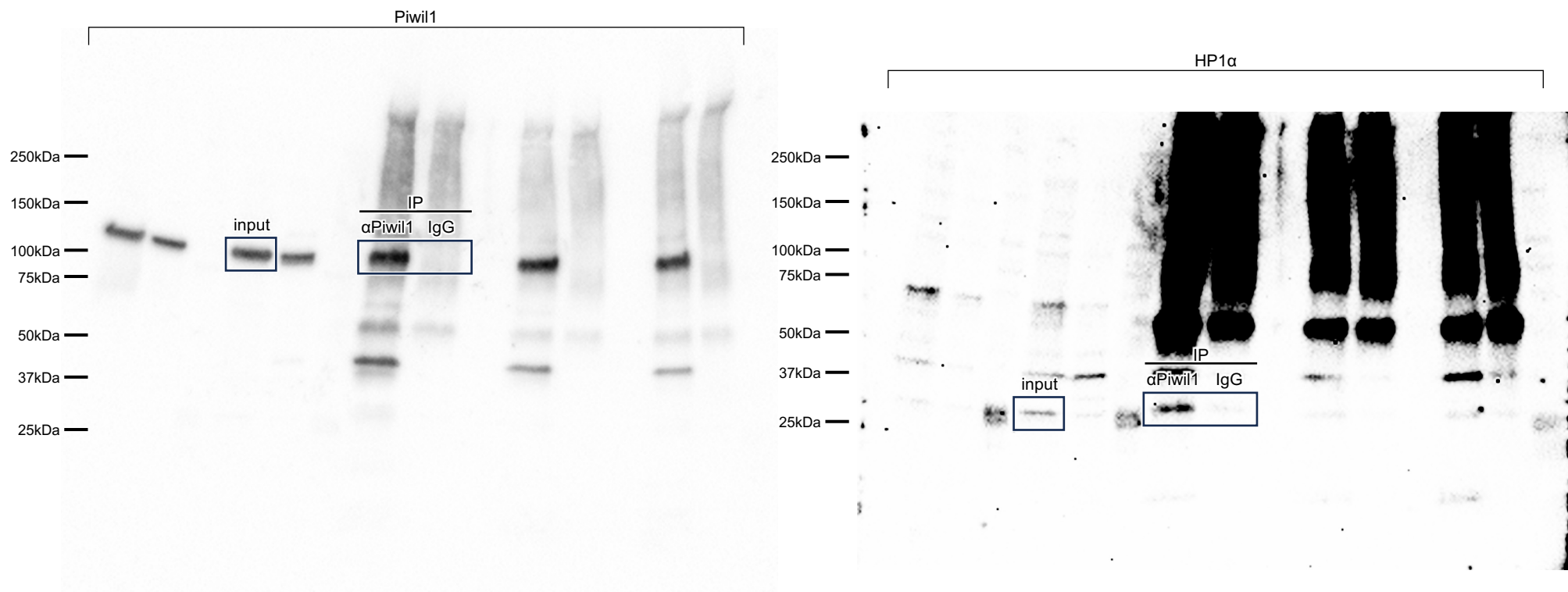

**Figure 6-source data 2.** Original membranes corresponding to Figure 6, panel I.

Supplement: Figure 6—source data 2. [file elife-104295-fig6-data2.zip › Figure 6-source data 2. PDF file containing original western blots for Figure 6I, indicating the relevant bands and treatments/Figure 6-source data 2 with the relevant bands labelled.pdf]

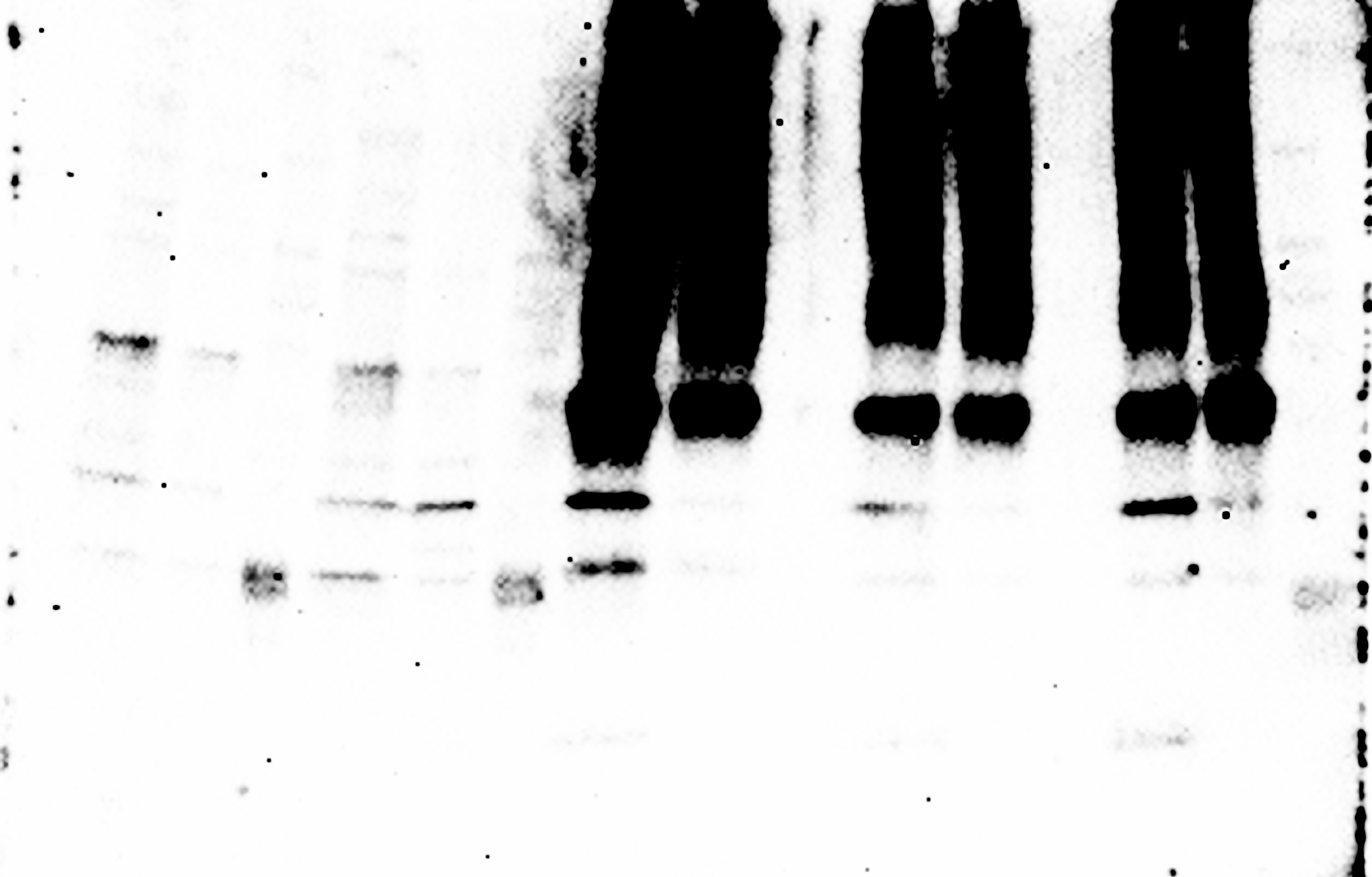

Supplement: Figure 6—source data 2. [file elife-104295-fig6-data2.zip › Figure 6-source data 2. PDF file containing original western blots for Figure 6I, indicating the relevant bands and treatments/The original file of HP1a-WB_input_Piwil1-IP.tif]

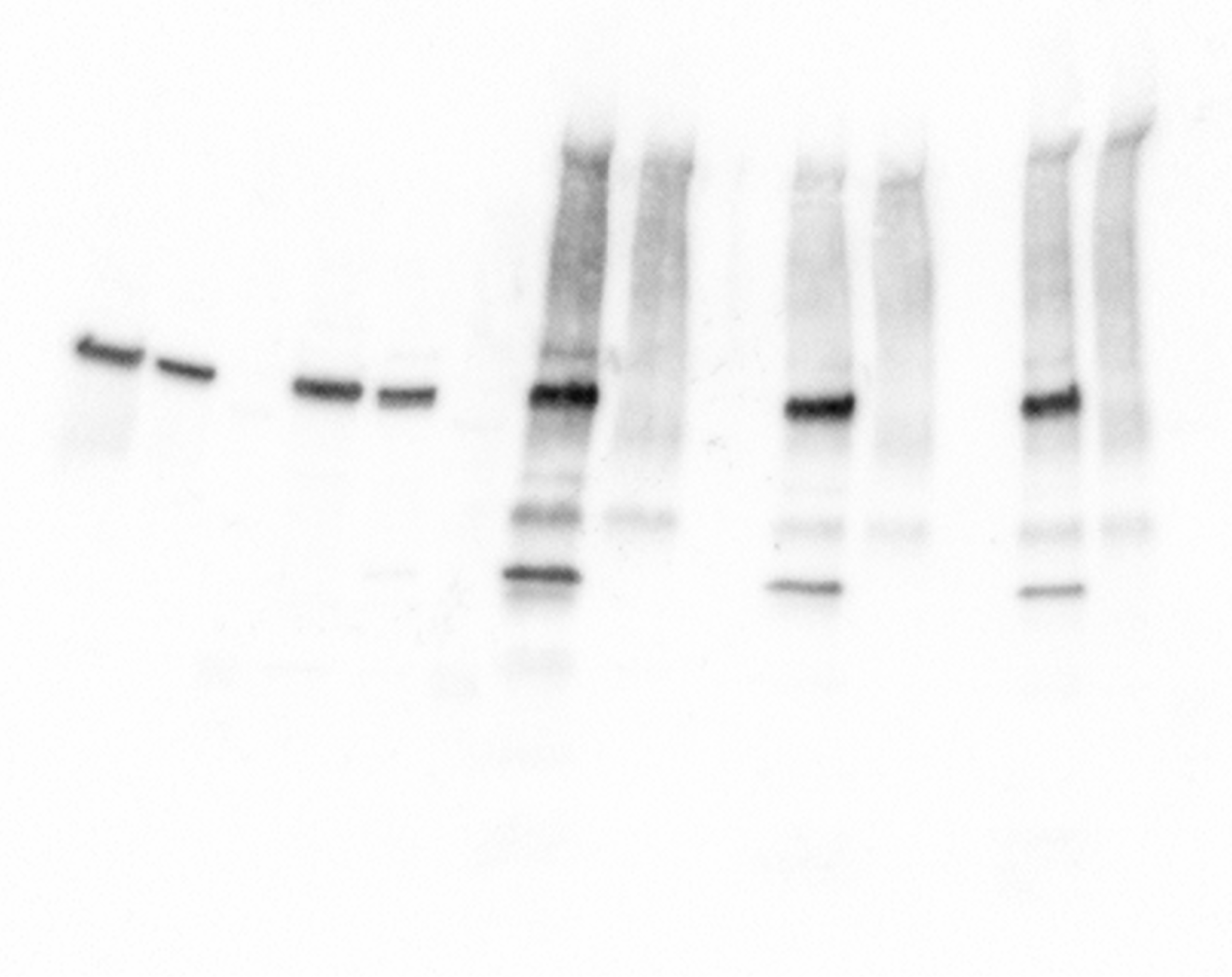

Supplement: Figure 6—source data 2. [file elife-104295-fig6-data2.zip › Figure 6-source data 2. PDF file containing original western blots for Figure 6I, indicating the relevant bands and treatments/The original file of Piwil1-WB_input_Piwil1-IP.tif]
